# Supplementary material for: FMRP regulates STAT3 mRNA localization to cellular protrusions and local translation to promote hepatocellular carcinoma metastasis
Source: Commun Biol. 2021 May 10;4:540. doi: 10.1038/s42003-021-02071-8 (PMC8110961; doi:10.1038/s42003-021-02071-8)
Supplement: Supplementary file 2 — Supplementary Information [file 42003_2021_2071_MOESM2_ESM.pdf]

Supplementary Information (SI) for:

FMRP regulates STAT3 mRNA localization to cellular protrusions and local translation to promote hepatocellular carcinoma metastasis

Materials and methods

GFP-MS2 system

The GFP-MS2 system contained two plasmids: pCR4-24×MS2SL-stable plasmid and p-GFP-MS2 plasmid. The p-GFP-MS2 plasmid expresses GFP-MS2 fusion protein, while pCR4-24×MS2SL-stable plasmid expresses STAT3 mRNA by placing 24*MS2 stem-loops in the 3′-UTR. After co-transfection of the two plasmids into cells, the GFP-MS2 protein will point the location of STAT3 mRNA.

Western blot analysis

The treated cells were lysed in RIPA buffer supplemented with 0.1% protease inhibitors and centrifuged at 14,800 rpm for 30 min. The supernatant was collected and the protein concentration was quantified by BCA Protein Assay Kit. Equal amount of proteins was separated on SDS-PAGE gel and electro-transferred to PVDF membrane (Millipore Corp, USA). The resulting membrane was blocked by 5% non-fat milk and 0.1% Tween-20 for 2 h at room temperature, followed by incubation with the primary antibody overnight at 4℃. Afterwards, the membranes were incubation with the corresponding HRP-conjugated secondary antibodies (1:1000) diluted in TBST and then washed extensively with TBST. The bands were visualized with an enhanced chemiluminescence (ECL) system. These data were quantified by densitometry. The Image J software was used to analyze the relative protein expression. Antibodies against STAT3 were from Cell Signaling Technology (87868) and Antibody against Fmrp was from Abcam (ab128095).

Boyden chamber assay for STAT3 isoform identification

Isolation of RNAs in cell protrusion was performed according to a reported method with slight change [1]. We coated cell culture inserts (BD) with collagen Type-I (10 μg/mL, Sigma C7661). Before RNAs were purified, HCCLM3 cells were cultured for 36 h. After RNA

extraction, RT-PCR was carried out.

Gel mobility-shift assay

CDNA fragment encoding the 3′ UTR of STAT3 mRNA was amplified and subcloned into pSP64 plasmid (Promega). ^{32}P-labeled RNA was in vitro generated by SP6 RNA polymerase from pSP64- 3′ UTR constructs. Transcribed RNA probes were purified after resolving in a 6% denaturing gel. RNA–protein gel-shift assays were performed at room temperature as described previously. Briefly, 5 μM recombinant FMRP was incubated with 1 μM RNA probe in 1× reaction buffer. The reaction buffer consists of 10 mM Tris (pH 7.5 at 20 ℃), 100 mM KCl, 1 mM EDTA, 0.1 mM DTT, 20 μM cyclic AMP, 5% (v/v) glycerol and 0.010 mg/mL BSA. The electrophoresis was carried out in a 10% (w/v) polyacrylamide (75:1 acrylamide:bisacrylamide) gel, cast in 40 mM Tris-acetate, 2.5 mM EDTA (pH 7.8 at 20 ℃) and run in 40 mM Tris-acetate, 2.5 mM EDTA (pH 7.8 at 20 ℃), supplemented with 20 μM cyclic AMP. The RNA–protein complexes were visualized by autoradiography.

RT-PCR and Quantitative PCR (Q-PCR)

For quantitative RT-PCR analysis, the total RNA was isolated using the Trizol method. The first strand cDNA synthesis was done using the Superscript RT (Invitrogen). Real time PCR was performed using the Light Cyclermachine (Roche biochemical sciences) and appropriate primers corresponding to STAT3, FMRP and GAPDH mRNAs. For semi-quantitative RT-PCR analysis, the total RNA was also isolated using the Trizol method. The first strand cDNA was synthesized and PCR was performed using appropriate primers according to the similar procedure.

Recombinant protein expression and purification

cDNA fragments coding for FMRP were amplified by PCR and cloned into pCold I (Biofeng). Recombinant protein contained a His6 tag at the N-terminus. The constructs were transformed into E. coli 10B strain (New England Biolabs) and expression of the recombinant proteins was induced with 0.5 mM IPTG for 4.5 h. Purification of recombinant proteins was performed according to the manufacturer’s protocol. The purity of the proteins was determined by SDS-PAGE and Coomassie Blue staining. Fractions containing pure proteins were pooled and extensively dialyzed against lysis buffer to remove maltose residues.

Supplementary Table 1. Sequences of oligonucleotides designed in this work.

Name		Primer sequence
shFMRP#1	Forward primer	5’CCGGTCCTATACCATTTCCGTAATTCTCGAGAATTACGGAAATGGTATAGGATTTTTG-3’
	Reverse primer	5’AATTCAAAAATCCTATACCATTTCCGTAATTCTCGAGAATTACGGAAATGGTATAGGA-3’
shFMRP#2	Forward primer	5’CCGGGCGTTTGGAGAGATTACAAATCTCGAGATTTGTAATCTCTCCAAACGCTTTTTG-3’
	Reverse primer	5’AATTCAAAAAGCGTTTGGAGAGATTACAAATCTCGAGATTTGTAATCTCTCCAAACGC-3’
Scramble/ shFMRP	Forward primer	5’CCGGCTATTCGAATAGTCTACAGAGCTCGAGCTCTGTAGACTATTCGAATAGTTTTTG-3’
	Reverse primer	5’AATTCAAAAACTATTCGAATAGTCTACAGAGCTCGAGCTCTGTAGACTATTCGAATAG-3’
ShSTAT3#1	Forward primer	5’-CCGGGCAAAGAATCACATGCCACTTCTCGAGAAGTGGCATGTGATTCTTTGCTTTTTG-3’
	Reverse primer	5’-AATTCAAAAAGCAAAGAATCACATGCCACTTCTCGAGAAGTGGCATGTGATTCTTTGC-3’
ShSTAT3#2	Forward primer	5’-CCGGGCACAATCTACGAAGAATCAACTCGAGTTGATTCTTCGTAGATTGTGCTTTTTG-3’
	Reverse primer	5’-AATTCAAAAAGCACAATCTACGAAGAATCAACTCGAGTTGATTCTTCGTAGATTGTGC-3’
Scramble/ shSTAT3	Forward primer	5’-CCGGTTCTCCGAACGTGTCACGTCTCGAGACGTGACACGTTCGGAGAATTTTTG -3’
	Reverse primer	5’-AATTCAAAAATTCTCCGAACGTGTCACGTCTCGAGACGTGACACGTTCGGAGAA -3’

Supplementary Table 2. Clinical characteristics of patients with liver cancer tissue specimens.

NO.	Sex	Age	Organ	Pathology diagnosis	T	N	M	TNM	Stage
1	M	63	Liver	Hepatocellular carcinoma	T3	N0	M1	T3N0M1	IV
2	F	53	Liver	Hepatocellular carcinoma	T1	N0	MO	T1N0M0	I
3	M	76	Liver	Hepatocellular carcinoma	T1	NX	M0	T1NXM0	I
4	M	45	Liver	Hepatocellular carcinoma	T2	N0	M1	T2N0M1	IV
5	M	72	Liver	Hepatocellular carcinoma	T2	N0	M1	T2N0M1	IV
6	M	27	Liver	Hepatocellular carcinoma	T3	N0	M3	T3N0M2	IV
7	M	49	Liver	Hepatocellular carcinoma	T2	N0	M0	T2N0M0	II
8	M	65	Liver	Hepatocellular carcinoma	T2	N0	M0	T2N0M0	II

NO.	Sex	Age	Liver	Diagnostic grading	T	N	M	TNM	Stage
1	M	60	Liver	Hepatocellular carcinoma	T2	N0	M0	T2N0M0	II
2	M	28	Liver	Gallbladder	T0	—	—	—	—
3	M	66	Liver	Hepatocellular carcinoma	T1	N0	M0	T1N0M0	I
4	M	59	Liver	Hepatocellular carcinoma	T3	NX	M0	T3NXM0	IIIA
5	M	63	Liver	Hepatocellular carcinoma	T3	NX	M0	T3NXM0	IIIA
6	M	56	Liver	Hepatocellular carcinoma	T2	N0	M0	T2N0M0	II
7	M	44	Liver	Hepatocellular carcinoma	T2	N1	M1	T2N1M1	IV
8	M	65	Liver	Hepatocellular carcinoma	T2	N0	M2	T2N0M2	IV
9	M	54	Liver	Hepatocellular carcinoma	T2	N0	M1	T2N0M1	IV
10	M	53	Liver	Hepatocellular carcinoma	T1	N0	M0	T1N0M0	I
11	F	77	Liver	Hepatocellular carcinoma	T1	N1	M0	T1N1M0	IIIC
12	M	36	Liver	Hepatocellular carcinoma	T1	N0	M0	T1N0M0	I
13	M	61	Liver	Hepatocellular carcinoma	T1	NX	M0	T1NXM0	I
14	M	62	Liver	Hepatocellular carcinoma	T2	N0	M0	T2N0M0	II
15	F	60	Liver	Hepatocellular carcinoma	T2	N0	M0	T2N0M0	II
16	F	58	Liver	Hepatocellular carcinoma	T2	N0	M0	T2N0M0	II
17	M	42	Liver	Hepatocellular carcinoma	T1	N0	M0	T1N0M0	I
18	M	58	Liver	Hepatocellular carcinoma	T1	NX	M0	T1NXM0	I
19	M	57	Liver	Hepatocellular carcinoma	T1	NX	M0	T1NXM0	I
20	M	55	Liver	Hepatocellular carcinoma	T2	N0	M0	T2N0M0	II
21	M	69	Liver	Hepatocellular carcinoma	T2	NX	M0	T2NXM0	II

Supplementary figures

a

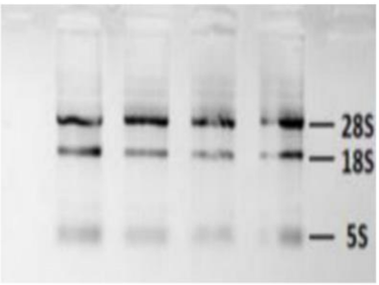

Supplementary Fig. 1 Observation of STAT3 mRNA localization in HCCLM3 protrusions. **a** Agarose gel electrophoresis analysis of mRNA isolated from cell protrusions. 28sRNA and 18sRNA bands are clearer, and 5s at the band is not clear, suggesting that RNA is not contaminated with DNA.

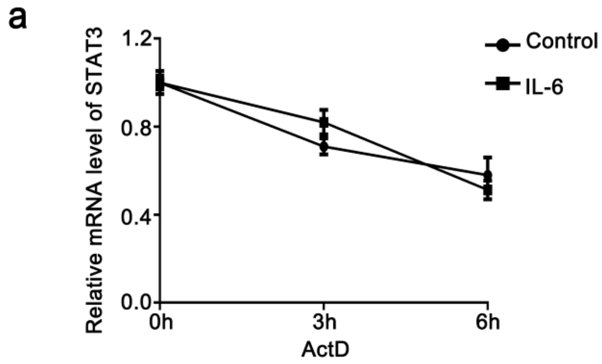

Supplementary Fig. 2 The 3'UTR of STAT3 mRNA is associated with its localization to cell protrusions. a STAT3 mRNA stability was determined by Q-PCR in control and IL-6-treated HCCLM3 cells using samples treated with ActinomycinD (ActD, transcriptional inhibitor) at the indicated time points. The values in the graphs represent the mean of three biologically independent experiments.

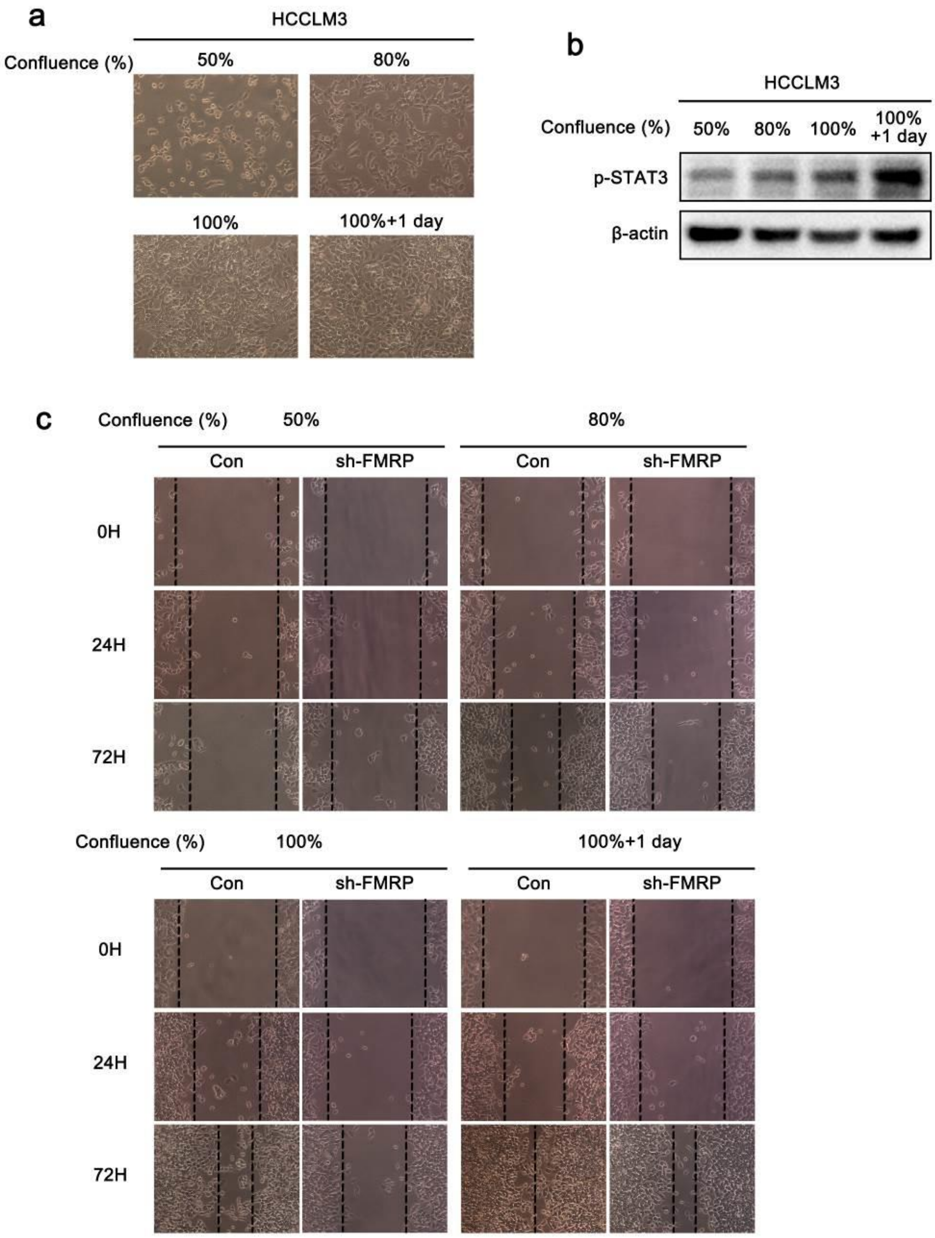

Supplementary Fig. 3 FMRP promotes cell migration independent of cell confluence. a HCCLM3 cells grown to the indicated confluences were photographed. **b** Cell density upregulates the level of STAT3-pTyr705. **c** Effect of FMRP knockdown on the rate of wound healing under different cell densities.

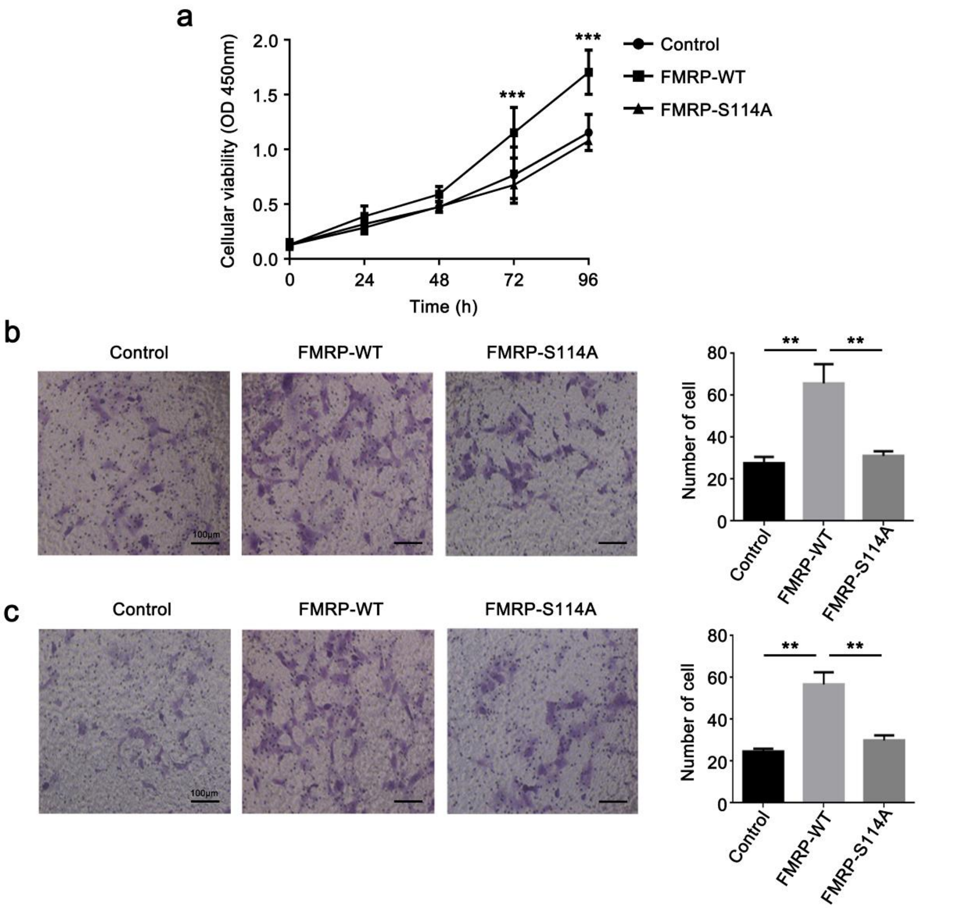

Supplementary Fig. 4 S114 is responsible for FMRP-mediated metastasis of HCC. a The proliferation ability of cells transfected with indicated plasmid was determined by CCK8 assay. **b, c** Transwell assay was performed to detect the migration (b) and invasion ability (c) of cells transfected with indicated plasmid. Scale bar: 100 μm. The values in the graphs represent the mean of three biologically independent experiments. Error bars represent ± s.d. **P* < 0.05, ***P* < 0.01, ****P* < 0.001 by 2-tailed Student's *t*-test.

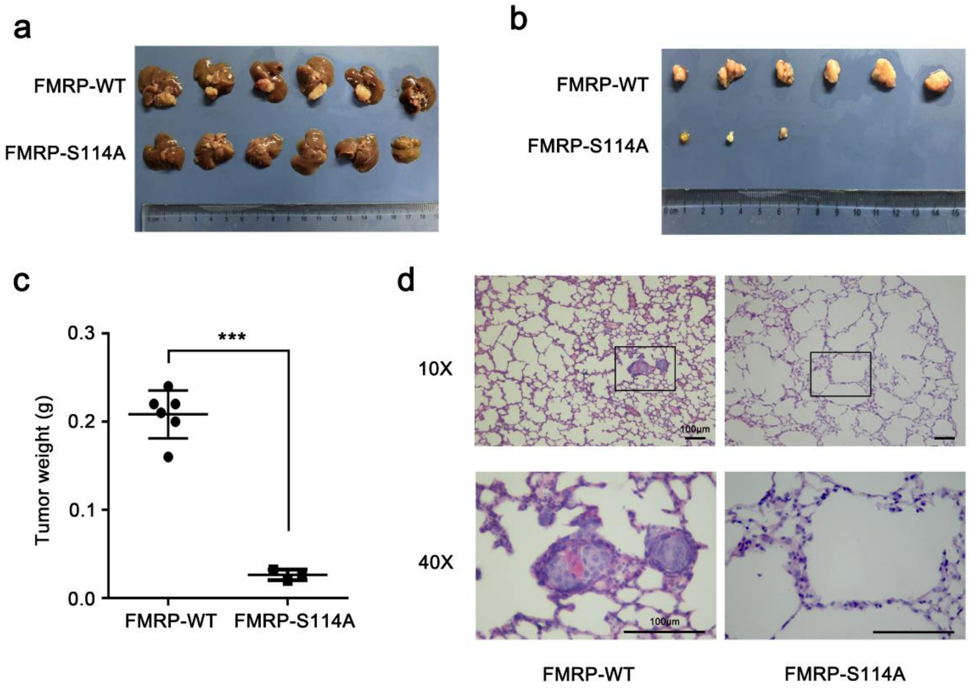

Supplementary Fig. 5 S114 is responsible for FMRP-mediated metastasis of HCC. a Livers injected with FMRP-S114-mutant cells to form xenograft tumors, accompanied by FMRP-WT groups. n=6 biologically independent samples (Three mice in FMRP-S114A group did not develop tumor).**b** Image of the xenograft tumors stripped from livers in nude mice. **c** Weights of xenograft tumors in two groups were measured. The values in the graphs represent the mean of six (/three) biologically independent samples. **d** Representative H&E staining of metastatic lung nodules at the two groups. Scale bar: 100 μm. Error bars represent ±s.d. **P* < 0.05, ***P* < 0.01, ****P* < 0.001 by 2-tailed Student's*t*-test.

Supplementary Fig. 6 Uncropped blots of figures 1-6.

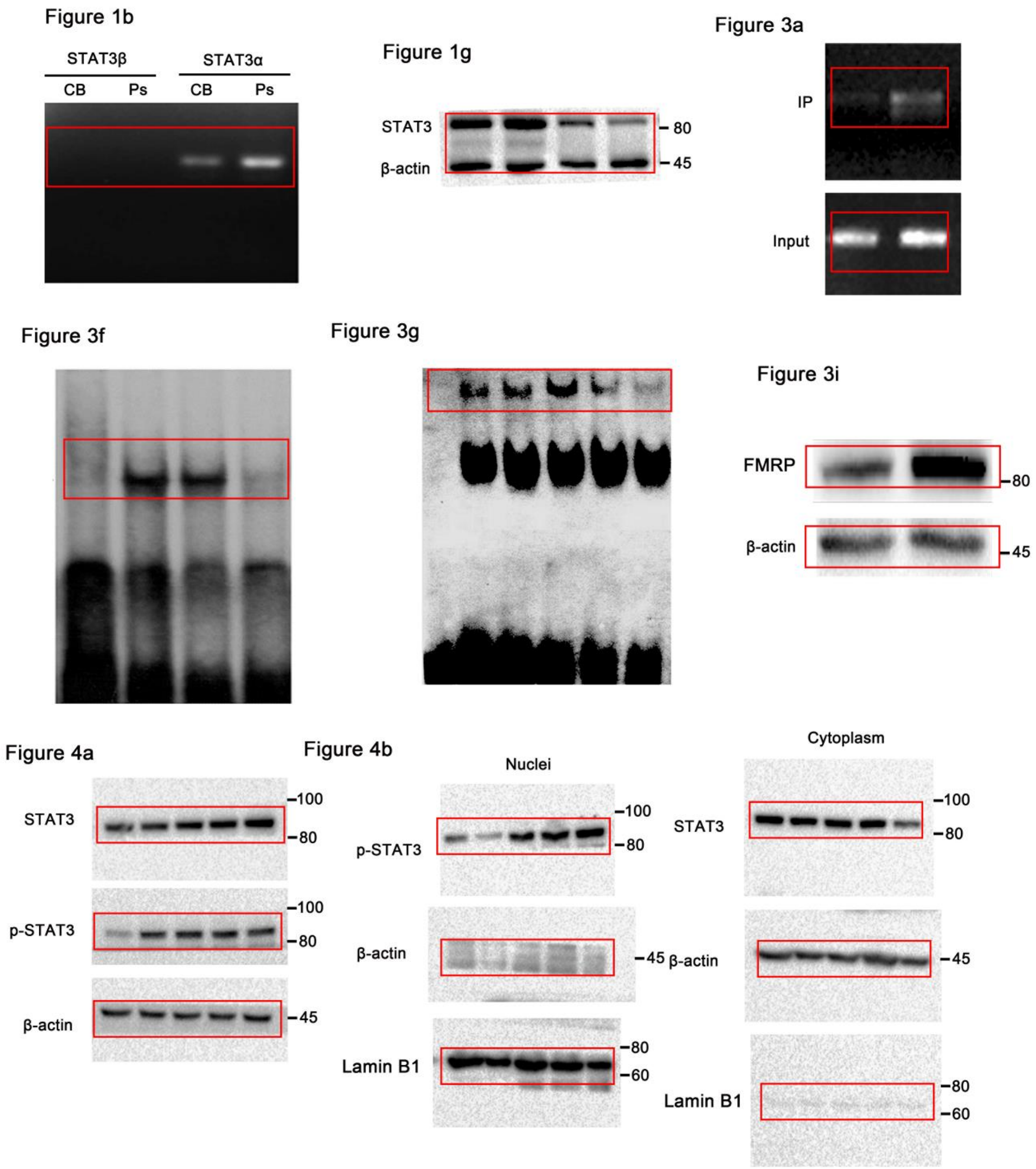

Figure 4d

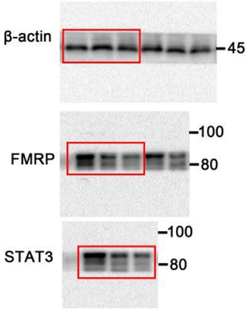

Figure 4f

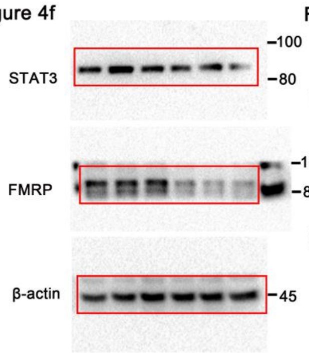

Figure 5c

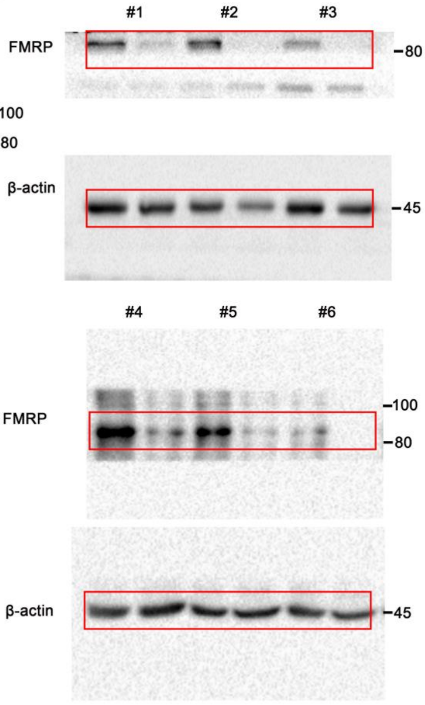

Figure 4g

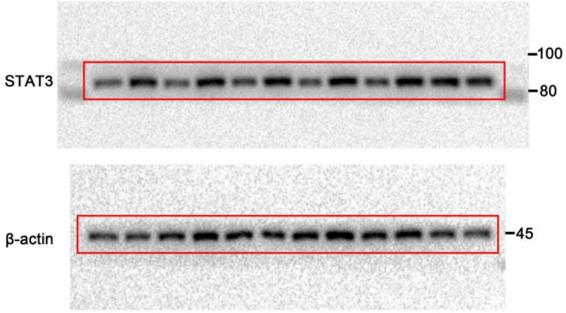

Figure 5d

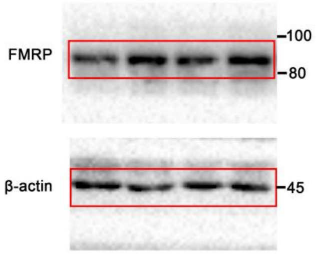

Figure 5f

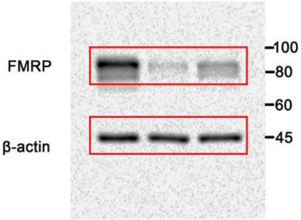

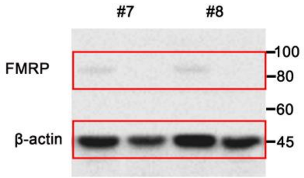

Figure 6e

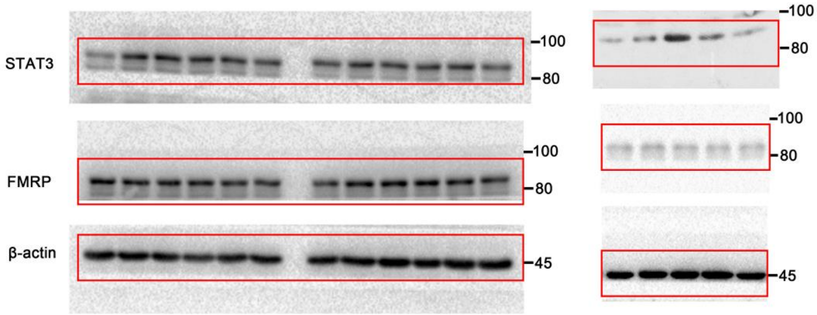

Supplementary references

1. Liu, Y.H., et al., Protrusion-localized STAT3 mRNA promotes metastasis of highly metastatic hepatocellular carcinoma cells in vitro. Acta Pharmacol Sin, 2016. 37(6): p. 805-13.
