# Supplementary material for: FMRP regulates STAT3 mRNA localization to cellular protrusions and local translation to promote hepatocellular carcinoma metastasis
Source: Commun Biol. 2021 May 10;4:540. doi: 10.1038/s42003-021-02071-8 (PMC8110961; doi:10.1038/s42003-021-02071-8)
Supplement: Supplementary file 3 — Description of Additional Supplementary Files [file 42003_2021_2071_MOESM3_ESM.pdf]

Description of Additional Supplementary Files

File name: Supplementary Data 1

Description: Source data underlying the graphs in the main figures.
