# Supplementary material for: FMRP regulates STAT3 mRNA localization to cellular protrusions and local translation to promote hepatocellular carcinoma metastasis
Source: Commun Biol. 2021 May 10;4:540. doi: 10.1038/s42003-021-02071-8 (PMC8110961; doi:10.1038/s42003-021-02071-8)
Supplement: Supplementary file 5 — Reporting Summary [file 42003_2021_2071_MOESM5_ESM.pdf]

Corresponding author(s): Zhifa Shen, Zaisheng Wu

Last updated by author(s): Feb 8, 2021

# Reporting Summary

Nature Research wishes to improve the reproducibility of the work that we publish. This form provides structure for consistency and transparency in reporting. For further information on Nature Research policies, see our Editorial Policies and the Editorial Policy Checklist.

## Statistics

For all statistical analyses, confirm that the following items are present in the figure legend, table legend, main text, or Methods section.

| n/a | Confirmed | |
|---|---|---|
| ☐ | ☒ | The exact sample size (*n*) for each experimental group/condition, given as a discrete number and unit of measurement |
| ☐ | ☒ | A statement on whether measurements were taken from distinct samples or whether the same sample was measured repeatedly |
| ☐ | ☒ | The statistical test(s) used AND whether they are one- or two-sided<br>*Only common tests should be described solely by name; describe more complex techniques in the Methods section.* |
| ☒ | ☐ | A description of all covariates tested |
| ☒ | ☐ | A description of any assumptions or corrections, such as tests of normality and adjustment for multiple comparisons |
| ☐ | ☒ | A full description of the statistical parameters including central tendency (e.g. means) or other basic estimates (e.g. regression coefficient) AND variation (e.g. standard deviation) or associated estimates of uncertainty (e.g. confidence intervals) |
| ☒ | ☐ | For null hypothesis testing, the test statistic (e.g. $F$, $t$, $r$) with confidence intervals, effect sizes, degrees of freedom and $P$ value noted<br>*Give P values as exact values whenever suitable.* |
| ☒ | ☐ | For Bayesian analysis, information on the choice of priors and Markov chain Monte Carlo settings |
| ☒ | ☐ | For hierarchical and complex designs, identification of the appropriate level for tests and full reporting of outcomes |
| ☒ | ☐ | Estimates of effect sizes (e.g. Cohen's *d*, Pearson's *r*), indicating how they were calculated |

*Our web collection on statistics for biologists contains articles on many of the points above.*

## Software and code

Policy information about availability of computer code

| | |
|---|---|
| Data collection | no software was used |
| Data analysis | Statistical significance was assessed using either Excel or GraphPad Prism8. Grey values for Western Blotting and immunofluorescence quantification were analyzed using Image J. |

For manuscripts utilizing custom algorithms or software that are central to the research but not yet described in published literature, software must be made available to editors and reviewers. We strongly encourage code deposition in a community repository (e.g. GitHub). See the Nature Research guidelines for submitting code & software for further information.

## Data

Policy information about availability of data

All manuscripts must include a data availability statement. This statement should provide the following information, where applicable:
- Accession codes, unique identifiers, or web links for publicly available datasets
- A list of figures that have associated raw data
- A description of any restrictions on data availability

We declare that the data supporting the findings of this study are available within the paper and its supplementary information files.

# Field-specific reporting

# Life sciences study design

All studies must disclose on these points even when the disclosure is negative.

| | |
|---|---|
| Sample size | No sample size was predetermined. Three or more independent samples were used to obtain results which were then used to perform statistical analysis. All sample sizes and the number of independent replicates are stated in figure legends. |
| Data exclusions | none. |
| Replication | The number of independent biological replicates are described in figure legends |
| Randomization | The samples were randomly allocated into experimental group. |
| Blinding | The investigators were blinded to group allocation during data collection and analysis. |

# Reporting for specific materials, systems and methods

We require information from authors about some types of materials, experimental systems and methods used in many studies. Here, indicate whether each material, system or method listed is relevant to your study. If you are not sure if a list item applies to your research, read the appropriate section before selecting a response.

## Materials & experimental systems

| n/a | Involved in the study |
|---|---|
| ☐ | ☒ Antibodies |
| ☐ | ☒ Eukaryotic cell lines |
| ☒ | ☐ Palaeontology and archaeology |
| ☐ | ☒ Animals and other organisms |
| ☒ | ☐ Human research participants |
| ☒ | ☐ Clinical data |
| ☒ | ☐ Dual use research of concern |

## Methods

| n/a | Involved in the study |
|---|---|
| ☒ | ☐ ChIP-seq |
| ☒ | ☐ Flow cytometry |
| ☒ | ☐ MRI-based neuroimaging |

## Antibodies

| | |
|---|---|
| Antibodies used | STAT3, Cell Signaling Technology, 8768S, D1A5; p-STAT3, Cell Signaling Technology, 9131S, D3A7; FMRP, abcam, ab17722, GR3266123-1; LaminB1, proteintech, 12987-1-AP; β-actin, proteintech, 20536-1-AP. |
| Validation | All antibodies that are commercially available have been tested for species reactivity and application by the manufacturers. |

## Eukaryotic cell lines

Policy information about cell lines

| | |
|---|---|
| Cell line source(s) | The cell lines were obtained from the Chinese Academy of Sciences. The Chinese Academy of Sciences purchased the cell lines from ATCC. |
| Authentication | The authentication of all cell lines used was performed by short tandem repeat (STR)-profiling. |
| Mycoplasma contamination | All cell lines tested negative for mycoplasma contamination. |
| Commonly misidentified lines (See ICLAC register) | No misidentified cell lines were used in this study. |

## Animals and other organisms

Policy information about studies involving animals; ARRIVE guidelines recommended for reporting animal research

| | |
|---|---|
| Laboratory animals | BALB/C-nu nude mice, male, 6 week |
| Wild animals | N/A |
| Field-collected samples | The study did not involve samples  collected from the field. |
| Ethics oversight | All animal experiments were approved by the Zhejiang Management Committee for Medical Laboratory Animal Sciences. |

Note that full information on the approval of the study protocol must also be provided in the manuscript.
